# Training for Marathons during a Marathon Pandemic: Effect of the COVID-19 Pandemic on Fitness among High-Level Nonelite Runners

**DOI:** 10.1155/2021/9682520

**Published:** 2021-10-05

**Authors:** Martin E. Matsumura, Bryan Martin, Thomas Matsumura, Ataul Qureshi

**Affiliations:** ^1^The Pearsall Heart Hospital, Geisinger Health System, Wilkes-Barre, PA, USA; ^2^Bucknell University, Lewisburg, PA, USA; ^3^Aultman Deuble Heart and Vascular Center, Canton, OH, USA

## Abstract

**Background:**

The COVID-19 pandemic has had a profound effect on all aspects of life, including physical fitness and well-being of the general population. In the present study, we assessed the effect of the pandemic on the subjective and objective fitness of high-level nonelite runners.

**Methods:**

The MASTERS Athletic Study is an ongoing survey of training and health habits of high-level nonelite runners, the majority of whom compete in marathons and other extreme endurance events. We invited participants to a web-based questionnaire regarding training and fitness during the COVID-19 pandemic. Comparisons were made between subjective and objective fitness as well as well as the relationship of prepandemic training volume and history of COVID-19 on change in fitness during the pandemic, using Mann–Whitney rank-sum tests and chi-square tests for nonparametric and categorical variables, respectively.

**Results:**

A total of 189 runners completed the survey, of whom 26 (13.8%) reported prior diagnosis with COVID-19. In terms of the subjective sense of fitness compared to before the pandemic, 49 (25.9%) reported they were less fit, 55 (29.1%) reported they were more fit, and 85 (45.0%) reported their fitness was unchanged. These assessments correlated well with objective measurement of training volume in MET-min/week. Runners with improved fitness at present had a lower calculated training volume before the pandemic versus those who reported unchanged or worsened fitness. There was no relationship between the report of prior COVID-19 and either subjective or objective measures of fitness.

**Conclusion:**

The COVID-19 pandemic has had a variable effect on the fitness of high-level nonelite runners. We found an inverse relationship between baseline training volume and the likelihood of runners reporting improved fitness and no relationship between a history of COVID-19 and change in fitness through the pandemic. Understanding the effect of the COVID-19 pandemic on athletic fitness will help guide strategies to maintain physical health and wellness through future public health crises.

## 1. Introduction

The COVID-19 pandemic and public health response to this crisis have dramatically changed multiple aspects of everyday life, including physical fitness and well-being. Several recent studies have revealed confirmatory evidence that the pandemic has had a significant effect on the overall fitness and resultant health status of a large number of individuals [1–5]. In addition to the general population, the running and endurance sports communities have not been excluded from the effects of the pandemic [6–9]. At the peak of the pandemic, social distancing regulations resulted in the cancellation of a majority of traditional competitive running events likely resulting in loss of motivation of many athletes to maintain high-level training habits [6]. Regulations regarding gyms and public fitness areas limited access to consistent training venues for many athletes. Finally, the resultant anxiety and depression related to social isolation may have had an impact on general physical and psychological well-being far beyond endurance fitness [10].

A number of studies have attempted to quantify the effect of the COVID-19 pandemic on both subjective and objective measures of physical fitness among regular exercisers, including a spectrum of athletes from casual to college-level athletes [6–8]. At least, one recent study quantified the effect of the pandemic on cardiorespiratory fitness, albeit among a group of adolescents rather than adult athletes [11]. This study documented slight reductions in VO2max in this cohort. The goal of the present study was to expand this knowledge base to a cohort of experienced and high-level nonelite runners. Specifically, our aims were to get a better understanding of the effect of the COVID-19 pandemic on the overall sense of fitness among these runners, to correlate this sense of fitness with the effect of the pandemic period on individual runners' training volume, and to assess the relationship of COVID-19 on these variables.

## 2. Methods

The current study was an online, secure, HIPAA-compliant survey regarding training habits, subjective fitness, and COVID-19 history among runners. An invitation to participate in the survey was sent to runners who had participated in the MASTERS Athletic Study beginning in 2014 [12]. The MASTERS Athletic Study was an internet-based survey of training and health characteristics among runners, primarily who train and compete at a relatively high level. As an example, over half of the cohort reported running a marathon or ultramarathon in the 5 years prior to the original survey, and two-thirds reported supplementing their training with speed work or interval training. The original survey gathered baseline information on these athletes and collected contact information for future surveys. An invitation to participate in the current study was e-mailed to the 1,900 individuals from the MASTERS database with active e-mail addresses on file on two occasions in February 2021.

The current study consisted of 25 questions comparing training volume and pace prior to the start of the COVID-19 pandemic in March 2020 and at the time of the survey, February 2021. The survey included questions about average weekly mileage, average training pace, and subjective sense of fitness at both time points. In addition, runners who were diagnosed with COVID-19 were asked additional questions regarding training and medical care during their illness. The survey is included in the Appendix.

Weekly training volume in MET-min/week was calculated from reported weekly training mileage and pace [13]. Changes in training volume were defined as current MET-min/week minus that reported before the pandemic. An increase or decrease of ≥10% from before the pandemic to the present was considered as a categorical increase or decrease in training volume. The 10% threshold in volume was chosen because this was felt to represent a clinically relevant change and because the volume changes among the cohort were not normally distributed. For subjective fitness, runners were asked to categorize their current fitness compared to before the pandemic on a scale from “much better” to “much worse.” A response of “much better” or “better” was categorized as “better” and “much worse” or “worse” was categorized as “worse” for analysis.

Statistical analysis was performed using Sigmastat 4.0 software (Systat, San Jose CA). Nonparametric data were compared using Mann–Whitney rank sum tests, and categorical data were compared using chi-square tests. For all comparisons, a *p* value less than 0.05 was considered significant.

The study has been approved by the Institutional Review Board of Geisinger Health System.

## 3. Results

A total of 189 runners (10% of those contacted by e-mail) completed the survey in full, with no omitted answers. 49 (25.9%) reported they were less fit, 55 (29.1%) reported they were more fit, and 85 (45.0%) reported their fitness was unchanged to the question “How is your fitness now compared to prior to the pandemic?” Comparisons between these 3 groups are shown in Table 1. There were no differences between reported subjective fitness and male/female sex, age range, years of running experience, or frequency or distribution of cross-training activities. In contrast, those runners who reported improved subjective fitness also reported significantly lower training volume prior to the pandemic vs. those who reported worsened fitness or no change (2046 MET-min/week for those with improved fitness vs. 3060 MET-min/week for those with worse fitness and 3024 MET-min/week for those without change, *p* < 0.005). We also calculated change in training volume from before the pandemic to the current time. We defined increased training volume as ≥10% increase in MET-min/week and decreased training volume as ≥10% decrease in MET-min/week over the pandemic period. When we compared reported subjective fitness to change in training volumes, we found that subjective fitness correlated very well with training volume changes, confirming that runners' subjective sense of their fitness correlated well with their actual reported training volume (Table 1, *p* < 0.001 comparing reported fitness with change in training volume during the pandemic).

Of the 189 runners who completed the survey, 26 (13.8%) reported being diagnosed with COVID-19. Characteristics of the infectious events reported by study respondents are summarized in Table 2. The majority of the respondents (23/26, 88.5%) reporting a COVID-9 were diagnosed within 3 months of the survey, and 85% of the respondents reported symptoms at the time of diagnosis. Half of the respondents reported continued symptoms at the time of the survey with the most common symptom being fatigue (9/26, 69.2%).

**Figure 1:**
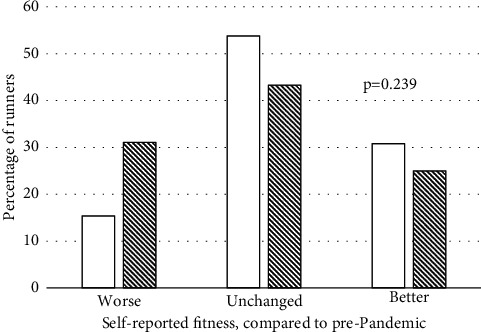
Distribution of current subjective fitness compared to that before the pandemic between runners not reporting a history of COVID-19 (solid bars) and runners reporting a history of COVID-19 (hatched bars).

**Figure 2:**
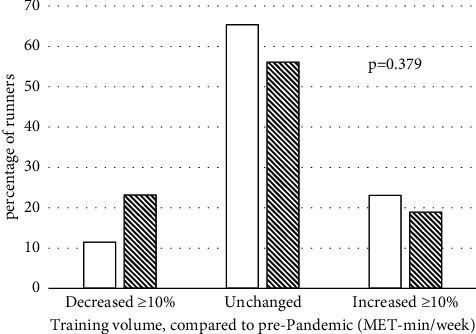
Distribution of changes in running volume in MET-min/week between runners not reporting a history of COVID-19 (solid bars) and runners reporting a history of COVID-19 (hatched bars).

We compared both subjective fitness and change in training volume during the pandemic across those runners who did versus those who did not report being infected with COVID-19 during the pandemic. Despite the history of COVID-19 and significant presence of continued symptomology among respondents, there were no significant differences in either the categorical distribution of self-reported fitness (Figure 1) or average weekly training volume (Figure 2) during the pandemic between those reporting COVID-19 and those who did not report being infected.

## 4. Discussion

A growing body of data is helping to shape our understanding of the effects of the COVID-19 pandemic and resultant public health responses on fitness, among both the general population and recreational athletes. The present study has several notable findings which add to this knowledge base. First, the effect of the COVID-19 pandemic on the fitness of high-level nonelite runners has been variable, with nearly equal respondents reporting an increase or decrease in the subjective sense of overall fitness. Second, this subjective sense of fitness correlated well with a more objective measure of running fitness, specifically weekly training volume defined by MET-min/week. Third, among variables we examined, we found that those runners who reported an increase in fitness over the pandemic period tended to have a lower baseline training volume versus those who reported no change or a worsening of fitness. Fourth, we found no significant relationship between runners who suffered COVID-19 and those who did not in terms of subjective fitness or training volume. These findings add to the current knowledge base concerning effects of the COVID-19 pandemic on runners and are, to the best of our knowledge, the first to examine a very high-volume cohort of athletes, with runners performing up to 6,000 MET-min/week, an exercise volume that is achieved by less than 15% of recreational athletes [14].

A number of studies have examined the effect of the COVID-19 pandemic and resultant restrictions on physical well-being. Several of these studies documented overall decreased physical activity among the general population following pandemic restrictions [1–5]. Recent studies examining the effect of the pandemic on endurance athletes have also noted significant effects. DeJong et al. examined running volume and motivation among 1147 runners worldwide during the pandemic. These runners reported an increase in weekly mileage but a decrease in both high-level workouts such as sprint intervals and a lower motivational score compared to before the pandemic [8]. A significant contributor to the decrease in motivation was the lack of a competitive environment during the pandemic. Notably, this sample represented relatively lower-volume runners compared to our study, and therefore, the finding of an overall increase in training volume in this cohort is similar to our finding of increased training volume and sense of overall fitness among those runners in our cohort with lower baseline training volume. Similarly, Fearnbach et al. examined changes in baseline physical activity among a large cohort of individuals with a mean exercise volume of approximately 1500 MET-min/week, a volume of exercise significantly less than that of participants in the present study [15]. Consistent with our results and those of DeJong, these authors found that individuals with the highest levels of physical activity at baseline reported the greatest decline during the pandemic. This consistent finding may be due to the fact that very-high-volume runners, i.e., those completing 50+ miles or 5,000+ MET-min per week, had a particularly difficult time maintaining their fitness due to the effects of social distancing and loss of competitive motivation during the pandemic. In contrast, Cloosterman et al. noted no differences in running behavior or volume among a large cohort of recreational runners in response to lockdown regulations in the Netherlands [9]. However, this cohort was similarly low volume, averaging less than 3 days of training per week, supporting the concept that low-volume runners suffered comparatively minimal effects on fitness in response to the COVID-19 pandemic.

The fact that we did not find a negative relationship between runners suffering COVID-19 and the sense of fitness or training volume is somewhat surprising, especially given the fact that most runners who reported a positive COVID-19 diagnosis indicated this event was a recent event and the majority also reported at least one continued symptom related to the illness [16–18]. Cloosterman et al. noted no association between running behavior and symptoms consistent with COVID-19 in a Dutch cohort; however, it should be noted that these athletes did not undergo confirmatory testing to assess COVID-19, and therefore, some or all of these infections may have been less-virulent community-acquired infections [9]. When comparing reports of fitness from these runners to those of runners not affected by COVID-19, the similarity suggests that the major determinants of fitness might be the overall impact on exercise of the social and public health regulations rather than the infections themselves. It is also possible that, in this cohort of high-level runners with excellent baseline fitness, the long-term effect of COVID-19 tends to be minimal. The number of respondents reporting COVID-19 was admittedly small, and results should be considered in this context.

Our study suffers from significant shortcomings, and the most obvious is that it is a relatively small and based on survey respondent data only. Recall survey data are prone to bias, and the possibility of bias affecting our results should be noted. The introduction of selection bias is a possibility given that runners who felt the COVID-19 pandemic had a substantial effect on their endurance fitness may have been particularly motivated to participate. We did not confirm either the test status of the runners who reported COVID-19 or the reported training volumes of all participants. Objective measures of exercise tolerance changes following COVID-19, such as results of cardiopulmonary stress testing, would obviously provide more concrete measures of the true effect of infection on fitness across a spectrum of athletes. Regardless, the present study represents, to the best of our knowledge, the first examination of the effects of the COVID-19 pandemic on high-level nonelite endurance athletes of a wide age range.

## 5. Conclusions

The present study demonstrates that the COVID-19 pandemic has had heterogeneous effects on the fitness of high-level nonelite runners, independent of whether or not these athletes had COVID-19. Athletes reporting the highest training volume before the pandemic suffered the greatest subjective and objective loss of fitness during the period of pandemic-related restrictions. Further study of the effect of the pandemic on overall fitness across a spectrum of baseline fitness will add to our understanding of how to direct efforts to maintain health and physical well-being in challenging times to come.

## Figures and Tables

**Table 1:** Comparison of respondents reporting their current fitness as “worse,” “better,” or “unchanged” versus the start of the COVID-19 pandemic.

	Compared to prior to the pandemic, my fitness is			*p*
	Worse	Unchanged	Better	
Total respondents (%)	49 (25.9)	85 (45.0)	55 (29.1)	n/a
Sex, male (%)	40 (81.6)	66 (77.6)	44 (80)	0.858
Age, range				
18–29	3 (6.1)	2 (2.4)	4 (7.3)	0.123
30–39	5 (10.2)	9 (10.6)	15 (27.3)	
40–49	12 (24.5)	21(24.7)	13 (23.6)	
50–59	15 (30.6)	23 (27.1)	9 (16.4)	
60+	14 (28.6)	30 (35.3)	14 (25.4)	
Running experience, years				
<5	2 (4.1)	2 (2.4)	5 (9.1)	0.371
6–15	16 (32.7)	23 (27.1)	21 (38.2)	
16–30	18 (36.7)	32 (37.6)	17 (30.9)	
>30	13 (26.5)	28 (32.9)	12 (21.8)	
Cross-training participation				
Cycling	33 (67.3)	51 (60.0)	36 (65.5)	0.653
Swimming	26 (53.1)	33 (38.8)	31 (56.3)	0.086
Other cross training	11 (22.4)	27 (31.8)	16 (29.1)	0.555
Running volume, prepandemic met-min/week, median (25%–75%)	3060 (2043–4016)	3024 (2040–4032)	2046 (2016–3060)	0.005
Change in training vol. from before the pandemic				
Decreased ≥10%	26 (53.1)	8 (9.4)	6 (10.9)	<0.001
No change	22 (44.9)	62 (72.9)	28 (50.9)	
Increased >10%	1 (2.0)	15 (17.6)	21 (38.2)	

**Table 2:** Details of runners reporting a history of COVID-19.

	*n* (%)
Total	26
When were you diagnosed with COVID-19?	
In the past month	10 (38.5)
1–3 months ago	13 (50)
4–6 months ago	1 (3.8)
>6 months ago	2 (7.7)
Were you symptomatic at the time of diagnosis?	
Yes	22 (84.6)
No	4 (15.4)
How much time did you take off from training?	
None	3 (11.5)
Less than 2 weeks	11 (42.3)
2–4 weeks	11 (42.3)
More than 4 weeks	1 (3.8)
Do you still have symptoms you attribute to the infection?	
Yes	13 (50)
What symptoms do you continue to experience?	
Fatigue	9 (69.2)
Weakness	6 (46.2)
Shortness of breath	4 (30.8)
Fast heart rate	4 (30.8)
Chest pain	2 (15.4)
Depression/lack of motivation	2 (15.4)
Other symptoms	2 (15.4)

## Data Availability

We will share our data upon request. They are stored online at our survey site.

## Supplementary Materials

Supplementary Materials Appendix: survey tool as accessed from the SurveyMonkey website.
