# Supplementary material for: Training for Marathons during a Marathon Pandemic: Effect of the COVID-19 Pandemic on Fitness among High-Level Nonelite Runners
Source: J Sports Med (Hindawi Publ Corp). 2021 Oct 5;2021:9682520. doi: 10.1155/2021/9682520 (PMC8492274; doi:10.1155/2021/9682520)
Supplement: Supplementary Materials — Appendix: survey tool as accessed from the SurveyMonkey website. [file 9682520.f1.pdf]

Copy of Study of the Effects of the COVID-19 Pandemic on Runners

Dear Runners,
On behalf of the MASTERS Athletic Survey we thank you for taking the time to participate in our survey.

We are interested in the effects of the COVID-19 pandemic on health and training habits of runners like you.

The study consists of 20 questions and should take less than 5 minutes to complete. You may leave your email address at the end of the survey if you would like a copy of the aggregate results.

Please note that this survey is for research purposes only; in no way does it constitute a medical evaluation or medical advice. Your answers will remain anonymous and will not be shared with any outside entities.

If you are over age 18 and run regularly we welcome you to participate in our survey.

This study has been approved by the institutional review board of Geisinger Health System. If you have questions please contact the study investigator at mmatsumura@geisinger.edu

1. What is your age?

- ◯ 18-24
- ◯ 25-29
- ◯ 30-34
- ◯ 35-39
- ◯ 40-44
- ◯ 45-49
- ◯ 50-54
- ◯ 55-59
- ◯ 60-64
- ◯ 65+

2. What is your gender identity?

- ◯ Female
- ◯ Male
- ◯ Prefer not to answer

3. For how many years have you been running for exercise and/or competitively?

- Less than 5 years
- 6-10 years
- 11-15 years
- 16-20 years
- 21-25 years
- 26-30 years
- >30 years

4. How many miles did you run in a typical week prior to the start of the COVID-19 pandemic (i.e. prior to March 2020)?

- <10
- 11-20
- 21-30
- 31-40
- 41-50
- >50

5. Since the start of the pandemic (i.e. March 2020) how many miles do you run in a typical week?

- <10
- 11-20
- 21-30
- 31-40
- 41-50
- >50

6. Prior to the COVID-19 pandemic what was your typical training pace (the pace at which you did most of your runs)?

- <6 minutes/mile
- 6-7 minutes/mile
- 7-8 minutes/mile
- 8-9 minutes/mile
- 9-10 minutes/mile
- 10-11 minutes/mile
- 11-12 minutes/mile
- >12 minutes/mile

7. Since the start of the pandemic (i.e. March 2020) what is your typical training pace?

- <6 minutes/mile
- 6-7 minutes/mile
- 7-8 minutes/mile
- 8-9 minutes/mile
- 9-10 minutes/mile
- 10-11 minutes/mile
- 11-12 minutes/mile
- >12 minutes/mile

8. Do you participate in other endurance activities besides running (check all that apply)?

- [ ] Cycling
- [ ] Swimming
- [ ] Weights
- [ ] Other cross training
- [ ] I do not do any training other than running

9. How would you compare your fitness currently vs. prior to the pandemic?

- Much better
- Better
- About the same
- Worse
- Much worse
- Unsure

10. Have you ever tested positive for COVID-19?

- Yes
- No

Copy of Study of the Effects of the COVID-19 Pandemic on Runners

11. Approximately when were you diagnosed with COVID-19?

- Within the past month
- In the past 1-3 months
- In the past 4-6 months
- More than 6 months ago

12. Did you have symptoms at or around the time you were diagnosed with COVID-19?

- Yes
- No

13. Did you require an emergency room visit or hospital admission due to your COVID-19 illness?

- Yes
- No

14. Are you back to your baseline level of running fitness following your illness?

- Yes
- No

15. Which of the following are preventing your return to normal running (check all that apply)?

- [ ] Shortness of breath
- [ ] chest pain
- [ ] Muscle weakness
- [ ] General fatigue
- [ ] Dizziness
- [ ] Fast heart rate
- [ ] Depression/lack of motivation
- [ ] Other
- [ ] None of the above, back to normal running

16. How much time did you take off from running after your COVID-19 diagnosis?

- no time off
- less than 2 weeks
- 2-4 weeks
- >4 weeks

17. Do you seek a doctor's advice regarding restarting running and exercise after testing positive for COVID-19?

- Yes
- No

18. How long did your doctor advise you to refrain from exercise after testing positive for COVID-19?

- ◯ Did not recommend taking time off
- ◯ <1 week
- ◯ 1-2 weeks
- ◯ 2-4 weeks
- ◯ >4 weeks
- ◯ I did not see a doctor or get an opinion

19. Did you get any of the following test prior to resuming running (check all that apply)?

- ☐ electrocardiogram (EKG)
- ☐ stress test
- ☐ echocardiogram (heart ultrasound)
- ☐ chest x ray
- ☐ none of these

20. What was your typical resting heart rate prior to contracting COVID-19 (i.e. your heart rate upon awakening in the morning)?

- ◯ <40 beats per minute
- ◯ 40-50 beats per minute
- ◯ 50-60 beats per minute
- ◯ 60-70 beats per minute
- ◯ 70-80 beats per minute
- ◯ 80-90 beats per minute
- ◯ 90-100 beats per minute
- ◯ >100 beats per minute
- ◯ I don't know

21. What is your typical resting heart rate since contracting COVID-19?

- ◯ <40 beats per minute
- ◯ 40-50 beats per minute
- ◯ 50-60 beats per minute
- ◯ 60-70 beats per minute
- ◯ 70-80 beats per minute
- ◯ 80-90 beats per minute
- ◯ 90-100 beats per minute
- ◯ >100 beats per minute
- ◯ I don't know

22. Do you notice any of the following regarding your heart rate since your COVID-19 diagnosis (check all that apply)?

- ☐ Heart rate increases more rapidly with exercise
- ☐ Heart rate slows more slowly after completion of exercise
- ☐ Heart rate increases randomly at rest
- ☐ None of the above

23. Thank you for participating! Your answers will remain confidential. Feel free to leave an email address if you would like a copy of the results of this study to be sent to you.
